# Effects of the Selective EP2 Receptor Agonist Omidenepag on Adipocyte Differentiation in 3T3-L1 Cells

**DOI:** 10.1089/jop.2019.0079

**Published:** 2020-04-07

**Authors:** Yasuko Yamamoto, Takazumi Taniguchi, Tomoaki Inazumi, Ryo Iwamura, Kenji Yoneda, Noriko Odani-Kawabata, Takeshi Matsugi, Yukihiko Sugimoto, Naveed K. Shams

**Affiliations:** ^1^ Research and Development Division, Santen Pharmaceutical Co., Ltd., Nara, Japan.; ^2^ Department of Pharmaceutical Biochemistry, Graduate School of Pharmaceutical Sciences, Kumamoto University, Kumamoto, Japan.; ^3^ Pharmaceuticals Research Laboratory, Pharmaceutical Division, Ube Industries, Ltd., Yamaguchi, Japan.; ^4^ Research and Development Division, Santen Pharmaceutical Co., Ltd., Osaka, Japan.; ^5^ Research and Development Division, Santen, Inc., Emeryville, California.

**Keywords:** EP2 agonist, omidenepag, FP agonist, adipogenesis, deepening of the upper eyelid sulcus

## Abstract

***Purpose:*** We aimed at comparing the effects of omidenepag (OMD) with those of prostaglandin F (FP) receptor agonists (FP agonists) on adipogenesis in mouse 3T3-L1 cells.

***Methods:*** To evaluate the agonistic activities of OMD against the mouse EP2 (mEP2) receptor, we determined cAMP contents in mEP2 receptor-expressing CHO cells by using radioimmunoassays. Overall, 3T3-L1 cells were cultured in differentiation medium for 10 days and adipocyte differentiation was assessed according to Oil Red O-stained cell areas. Changes in expression levels of the adipogenic transcription factors *Pparg*, *Cebpa*, and *Cebpb* were determined by using real-time polymerase chain reaction (PCR). OMD at 0.1, 1, 10, and 40 μmol/L, latanoprost free acid (LAT-A) at 0.1 μmol/L, or prostaglandin F_2α_ (PGF_2α_), at 0.1 μmol/L were added to cell culture media during adipogenesis. Oil Red O-stained areas and expression patterns of transcription factor targets of OMD or FP agonists were compared with those of untreated controls.

***Results:*** The 50% effective concentration (EC_50_) of OMD against the mEP2 receptor was 3.9 nmol/L. Accumulations of Oil Red O-stained lipid droplets were observed inside control cells on day 10. LAT-A and PGF_2α_ significantly inhibited the accumulation of lipid droplets; however, OMD had no effect on this process even at concentrations up to 40 μmol/L. LAT-A and PGF_2α_ significantly suppressed *Pparg*, *Cebpa*, and *Cebpb* gene expression levels during adipocyte differentiation. Conversely, OMD had no obvious effects on the expression levels of these genes.

***Conclusions:*** A selective EP2 receptor agonist, OMD, did not affect the adipocyte differentiation in 3T3-L1 cells, whereas FP agonists significantly inhibited this process.

## Introduction

Glaucoma is a neurodegenerative optical neuropathy that is characterized by the loss of retinal ganglion cells and their axons and is a leading cause of irreversible vision loss.^1,2^ Intraocular pressure (IOP) reduction is currently the only evidence-based treatment strategy for glaucoma. Lowering of IOP using prostaglandin F (FP) receptor agonists (FP agonists), such as latanoprost, tafluprost, travoprost, and bimatoprost, is the current standard of care for patients with glaucoma and ocular hypertension.^3^ However, prostaglandin-associated periorbitopathy (PAP) has been reported in FP agonist-treated patients with glaucoma.^4,5^ PAP affects patient care in many ways, such as difficulty in IOP measurement, difficulty during surgery, and cosmetic concerns.^4,5^ PAP is more frequent and more severe in bimatoprost users than in those using other FP agonists,^6^ and it causes deepening of the upper eyelid sulcus (DUES) and pigmentation of the iris and skin surrounding the eye lid.^4^ DUES is considered a cosmetic adversity of FP agonist treatments.^7–9^ The long-term use of latanoprost has also been considered causative of DUES in case studies of patients with glaucoma.^10,11^ Moreover, recent investigations suggest that DUES is induced by atrophy of orbital fat.^12^

Prostaglandin E_2_ (PGE_2_) acts on a group of G-protein-coupled receptors, and the subtypes EP1, EP2, EP3, and EP4 have been shown to respond to PGE_2_.^13,14^ Although PGE_2_ has been shown to potently reduce IOP in a previous study, it was associated with adverse effects (AEs), such as flares of anterior chambers.^15^ Thus, PGE_2_ receptor agonists that reduce ocular hypertension with little or no AEs are being investigated globally.^16^

We are currently developing omidenepag isopropyl (OMDI) as a new IOP-lowering ophthalmic solution.^17,18^ This agent is a prodrug of the selective non-prostaglandin EP2 receptor agonist and was launched as a treatment for glaucoma and ocular hypertension first in Japan in 2018. OMDI is hydrolyzed by esterases to omidenepag (OMD) during corneal penetration, and the IOP-lowering effects of this drug are associated with increased outflow facility and uveoscleral outflow.^19^ In a previous clinical study, topical applications of 0.002% OMDI significantly reduced IOP in patients with glaucoma, and all of the associated ocular AEs were mild in severity.^20^

FP agonists, including latanoprost, inhibit adipogenesis by stimulating FP receptor in 3T3-L1 cells.^21^ Prostaglandin F_2α_ (PGF_2α_) also inhibits adipocyte differentiation by binding the FP receptor.^22^ Hence, DUES due to current anti-glaucoma FP agonists likely follows inhibition of adipogenesis around the eyelid, followed by atrophy of orbital fat. Because the effects of the EP2 agonist OMD on adipocyte differentiation have not yet been demonstrated, we monitored adipocyte differentiation in 3T3-L1 cells treated with the pharmacologically active form of OMDI, and we made comparisons with the effects of other FP agonists.

## Methods

### Culture of CHO cells expressing mouse EP2 receptor and cAMP assays

CHO cells stably expressing mouse EP2 (mEP2) receptor^23^ and mock-CHO^24^ were cultured in 24-well plates at 2 × 10^5^ cells/well. Cells were then preincubated for 10 min at 37°C in N-(2-hydroxyethyl)piperazine-N′-2-ethanesulfonic acid (HEPES)-buffered saline containing 10 μmol/L indomethacin, and reactions were started by the addition of OMD at 0.1, 1, 10, 100, 1,000, and 10,000 nmol/L, which was provided by Ube Industries, Ltd. (Yamaguchi, Japan). After incubation for 10 min at 37°C, reactions were terminated by the addition of 10% trichloroacetic acid. Subsequently, cAMP concentrations in cells were measured by using radioimmunoassays according to a cAMP assay system (Cyclic AMP kit Yamasa, Yamasa Corporation, Chiba, Japan).

### Culture and differentiation of 3T3-L1 cells into adipocytes

Overall, 3T3-L1 cells were purchased from ATCC and were cultured in accordance with the supplier's instruction manual. Briefly, 3T3-L1 cells were grown to confluence in 24-well plates containing Dulbecco's modified Eagle's medium (high-glucose; Nacalai Tesque, Kyoto, Japan) supplemented with 10% calf serum (CS; Gibco, NY). Differentiation was initiated by culturing cells in differentiation medium containing 10% CS, 0.5 mmol/L isobutylmethylxanthine, 2.5 μmol/L dexamethasone, and 10 μg/mL insulin (AdipoInducer Reagent; Takara, Shiga, Japan). After 2 days of culture, media were replaced with adipocyte growth medium containing 10% CS and 10 μg/mL insulin and were exchanged every 2 or 3 days for an additional 8 days. Latanoprost free acid (LAT-A, 0.1 μmol/L), PGF_2α_ (0.1 μmol/L), from Cayman Chemical Co. (MI), or OMD (0.1, 1, 10, and 40 μmol/L) were added to adipocyte differentiation medium and growth medium (Table 1).

**Table 1.** Experimental Groups

Group	Final concentration of compound (μmol/L)	Culture medium	
		Day 0 to day 2	Day 2 to day 10
Control	0	Differentiation medium containing 0.1% DMSO	Growth medium containing 0.1% DMSO
0.1 μmol/L OMD	0.1	OMD 0.1 μmol/L differentiation medium containing 0.1% DMSO	OMD 0.1 μmol/L growth medium containing 0.1% DMSO
1 μmol/L OMD	1	OMD 1 μmol/L differentiation medium containing 0.1% DMSO	OMD 1 μmol/L growth medium containing 0.1% DMSO
10 μmol/L OMD	10	OMD 10 μmol/L differentiation medium containing 0.1% DMSO	OMD 10 μmol/L growth medium containing 0.1% DMSO
40 μmol/L OMD	40	OMD 40 μmol/L differentiation medium containing 0.1% DMSO	OMD 40 μmol/L growth medium containing 0.1% DMSO
0.1 μmol/L LAT-A	0.1	LAT-A 0.1 μmol/L differentiation medium containing 0.1% DMSO	LAT-A 0.1 μmol/L growth medium containing 0.1% DMSO
0.1 μmol/L PGF_2α_	0.1	PGF_2α_ 0.1 μmol/L differentiation medium containing 0.1% DMSO	PGF_2α_ 0.1 μmol/L growth medium containing 0.1% DMSO

DMSO, dimethyl sulfoxide; LAT-A, latanoprost free acid; OMD, omidenepag; PGF_2α_, prostaglandin F_2α_.

### Cell viability assays

Cell viability was determined by using MTS assays (CellTiter 96^®^ Aqueous One Solution Reagent; Promega, WI) with a Benchmark Plus Microplate Reader (Bio-Rad, CA) at an absorbance wavelength of 490 nm. On day 10 after initiation of differentiation, MTS reagent was added to each well and incubated for 30 min to measure the absorbance. The average absorbance of wells without cells was subtracted from absorbance values of cells. Data are presented as percentages of viable cells relative to controls.

### Oil Red O staining

Oil Red O staining was performed by using a Lipid assay kit (Cosmo Bio, Sapporo, Japan) in accordance with the manufacturer's instructions. Briefly, differentiated 3T3-L1 cells were washed in Dulbecco's phosphate-buffered saline (D-PBS), fixed in 10% formalin neutral buffer solution (FUJIFILM Wako Pure Chemical Corporation, Osaka, Japan) for 1 h, and stained with Oil Red O for 1 h. Oil Red O-stained cells were observed by using a microscope (IX70; Olympus, Tokyo, Japan). Six stained areas per well were then measured by using Win ROOF^®^ ver. 5.8 (MITANI Corporation, Tokyo, Japan). Cell areas that were stained with LAT-A, PGF_2α_, or OMD were expressed as percentages of those in untreated control cultures.

### Gene expression analysis

Total RNA was isolated from 3T3-L1 cells on indicated differentiation days by using RNeasy mini Kits (QIAGEN, Hilden, Germany and Venlo, Netherlands). Quantities and qualities of isolated RNA were evaluated by using NanoDrop™ (Thermo Fisher Scientific, Inc., MA). After dilution in RNase-free water, 20 ng/μL RNA samples were immediately reverse transcribed into cDNA by using PrimeScript™ RT Master Mix reagent Kits (Takara, Shiga, Japan) in accordance with the manufacturer's instructions. Primers for mouse peroxisome proliferator-activated receptor γ (PPARγ; *Pparg*), CCAAT/enhancer-binding protein α (C/EBPα; *Cebpa*), and *β* (C/EBPβ; *Cebpb*) (Takara) were used to quantify gene expression levels with QuantiFast™ SYBR^®^ Green PCR Kits (QIAGEN, Hilden, Germany) as described by the manufacturer (Table 2). Briefly, cDNA was amplified in the presence of SYBR Green PCR Master Mix in final reaction volumes of 20 μL per well by using a 7500 Fast Real-Time PCR System (Thermo Fisher Scientific, Inc., and Life Technologies, Inc., CA). Relative expression levels were calculated by using the standard ΔΔCt method with 7500 Fast System SDS Software Version1.4 software (Applied Biosystems, CA). The housekeeping gene *Gapdh* was used as an internal control.

**Table 2.** Primer Sets Used in This Study

Gene name	Forward	Reverse
*Gapdh*	5′-TGTGTCCGTCGTGGATCTGA-3′	5′-TTGCTGTTGAAGTCGCAGGAG-3′
*Pparg*	5′-GGAGCCTAAGTTTGAGTTTGCTGTG-3′	5′-TGCAGCAGGTTGTCTTGGATG-3′
*Cebpa*	5′-CAGCTTACAACAGGCCAGGTTTC-3′	5′-GCTGGCGACATACAGTACACACAA-3′
*Cebpb*	5′-ACCGGGTTTCGGGACTTGA-3′	5′-CCCGCAGGAACATCTTTAAGTGA-3′

### Statistical analysis

Fifty percent effective concentrations (EC_50_) were calculated by using EXSUS (version 8.0; CAC Croit Co., Tokyo, Japan). Data were expressed as mean ± SEM and were statistically analyzed by using EXSUS. Differences between drug treatment and control groups were identified by using Student's *t*-test, Wilcoxon test, Dunnett's test, or Steel test, and were considered significant when *P* < 0.05.

## Results

### mEP2 receptor agonist activity of OMD

**FIG. 1.**
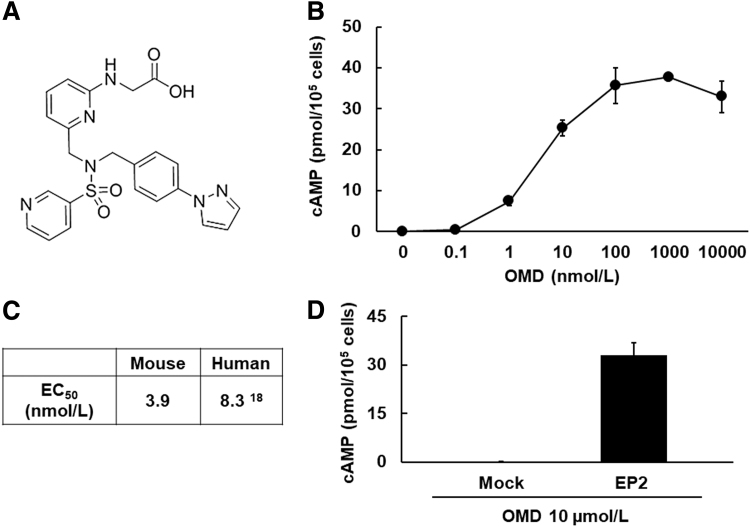
Agonistic activity of OMD toward the mouse EP2 receptor. **(A)** Structure of OMD. **(B)** Representative results of cAMP production of OMD. CHO cells expressing mEP2 receptor (2 × 10^5^ cells/well) were stimulated with the indicated concentrations of OMD for 10 min at 37°C. Changes in cAMP concentrations were determined by using radioimmunoassays. **(C)** Three independent experiments from 3 data points were performed, and the EC_50_ were calculated for the mEP2 receptor. **(D)** Results of cAMP production of OMD in CHO cells expressing mEP2 receptor and mock-CHO cells. mEP2 and mock-CHO cells (2 × 10^5^ cells/well) were stimulated with the indicated concentrations of OMD for 10 min at 37°C. Changes in cAMP concentrations were determined by using radioimmunoassays. EC_50_, 50% effective concentration; mEP2, mouse EP2; OMD, omidenepag.

To investigate the agonistic activity of OMD toward the mEP2, we measured cAMP concentrations in CHO cells after treatments with OMD by using radioimmunoassays. OMD treatments (Fig. 1A) promoted cAMP production dose dependently (Fig. 1B), and they had an EC_50_ value of 3.9 ± 0.49 nmol/L for the mEP2 receptor (*n* = 3) (Fig. 1C). The EC_50_ value was calculated from 3 independent replicates. In addition, OMD did not promote the formation of cAMP in CHO cells in which the mEP2 receptor was not stably expressed (Fig. 1D).

### Viability of 3T3-L1 cells after treatments with OMD or FP agonists

**FIG. 2.**
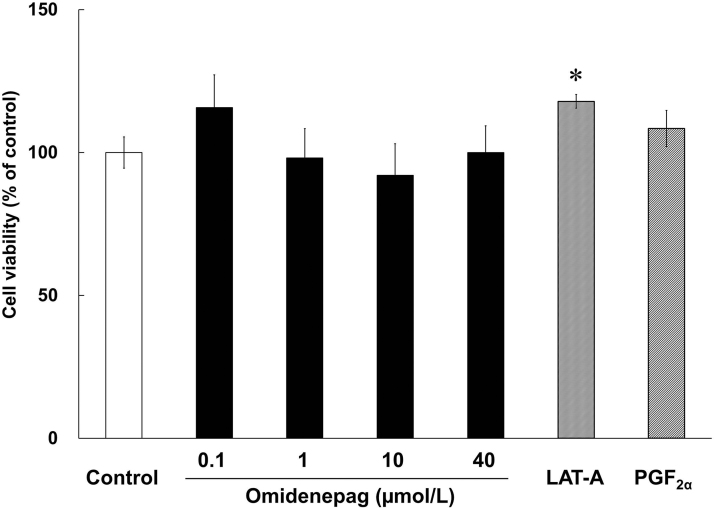
Viability of treated 3T3-L1 cells. Cell viability was assessed in 3T3-L1 cells during days 0–10 of differentiation in the presence of test compounds. Bars represent percentages of viable cells relative to those in control wells. *P < 0.05, compared with control (Student's *t*-test).

After 10 days of differentiation, relative to control, the cell viability of cells treated with OMD concentrations of 0.1, 1, 10, and 40 μmol/L was 115.7% ± 11.47% (*P* = 0.63), 98.1% ± 10.28% (*P* = 0.99), 92.0% ± 11.07% (*P* = 0.93), and 100.0% ± 9.39% (*P* = 1.00), respectively. In addition, after treatments with LAT-A and PGF_2α_, the cell viability was 117.9% ± 2.39% (*P* < 0.05) and 108.4% ± 6.32% (*P* = 0.37), respectively. OMD and PGF_2α_ did not affect cell viability in comparison with the control, whereas LAT-A treatments led to increases in cell viability (Fig. 2).

### Effects of OMD and FP agonists on adipogenesis

**FIG. 3.**
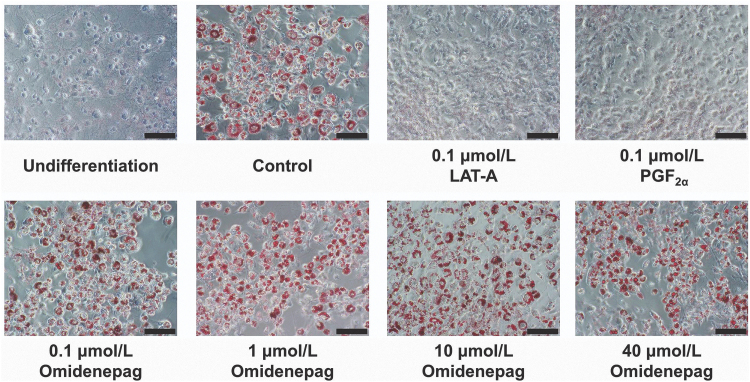
Representative photographs of Oil Red O staining. Oil Red O staining analyses were performed in 3T3-L1 cells treated with test compounds during days 0–10 of differentiation. Adipogenesis in 3T3-L1 cells was assessed by using Oil Red O staining on day 10. Scale bar = 100 μm.

**FIG. 4.**
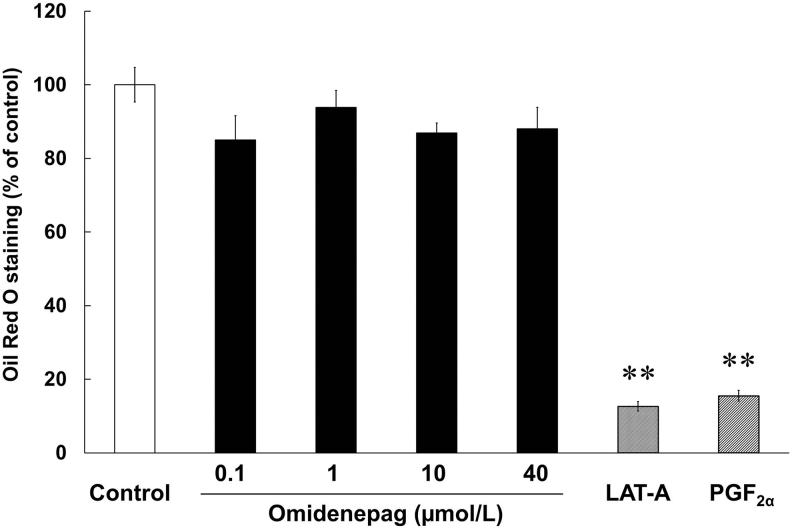
Different effects of OMD and FP agonists on adipogenesis. Oil Red O staining analyses were performed in 3T3-L1 cells after treatments with test compounds during days 0–10 of differentiation. **P < 0.01, compared with control (Student's *t*-test). FP, prostaglandin F.

To evaluate the effects of OMD and FP agonists on adipogenesis, we performed Oil Red O staining of lipid droplets in 3T3-L1 cells after differentiation for 10 days. Marked increases in numbers of lipid droplets confirmed preadipocyte differentiation in control, as shown in previous report^21^ (Fig. 3). Oil Red O staining patterns also differed significantly between cells treated with OMD and FP agonists (Fig. 3). Relative to control cells, stained areas comprised 12.6% ± 1.3% and 15.5% ± 1.5% of cell areas after treatments with LAT-A and PGF_2α_, respectively. In addition, after treatments with OMD at 0.1, 1, 10, and 40 μmol/L, stained areas comprised 85.0% ± 6.6%, 93.9% ± 4.5%, 86.9% ± 2.7%, and 88.0% ± 5.9% of cell areas, respectively (Fig. 4). These data show that OMD treatments had no significant effects on adipogenesis. In contrast, the FP agonists LAT-A and PGF_2α_ significantly inhibited adipogenesis in 3T3-L1 cells.

### Adipogenic transcription factor expression during adipogenesis

**FIG. 5.**
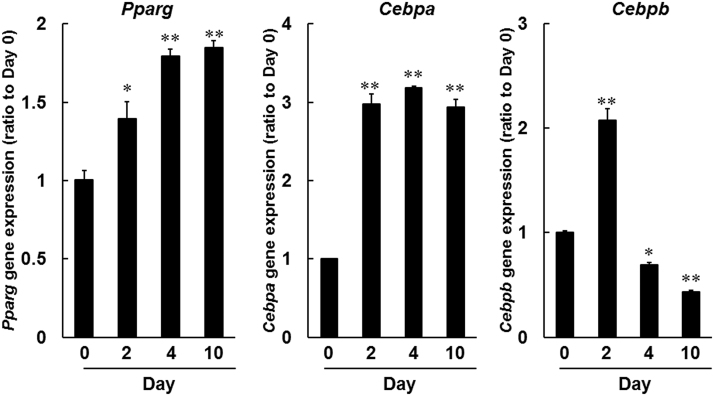
Gene expression analyses of adipogenic transcription factors during adipogenesis. *Pparg*, *Cebpa*, and *Cebpb* mRNA expression levels were determined by using real-time PCR, and comparisons were made with expression levels on day 0. *P < 0.05, **P < 0.01, compared with day 0 (Dunnett test). PCR, polymerase chain reaction.

The adipogenic transcription factors *Pparg* and *Cebp* sequentially stimulate genetic changes that result in differentiation.^25,26^ Herein, we confirmed these changes in transcription factor expression during adipogenesis in 3T3-L1 cells by using quantitative real-time polymerase chain reaction (PCR) analyses of *Pparg*, *Cebpa*, and *Cebpb* mRNAs. In time course experiments (Fig. 5) in differentiating 3T3-L1 cells, *Pparg* mRNA expression levels were increased by 1.4-fold at day 2, and by 1.8-fold at days 4 and 10. *Cebpa* expression was similarly increased by 2.9-fold at days 2 and 10, and by 3.2-fold at day 4, and that of *Cebpb* was increased by 2.1-fold by day 2. These results show early increases in *Pparg* and *Cebpa* mRNA expression levels during adipogenesis in 3T3-L1 cells. *Cebpb* was also transiently induced during the early stages of differentiation, as shown in previous studies.^25,26^

### Effects of OMD and FP agonists on mRNA expression of adipogenic transcription factors

**FIG. 6.**
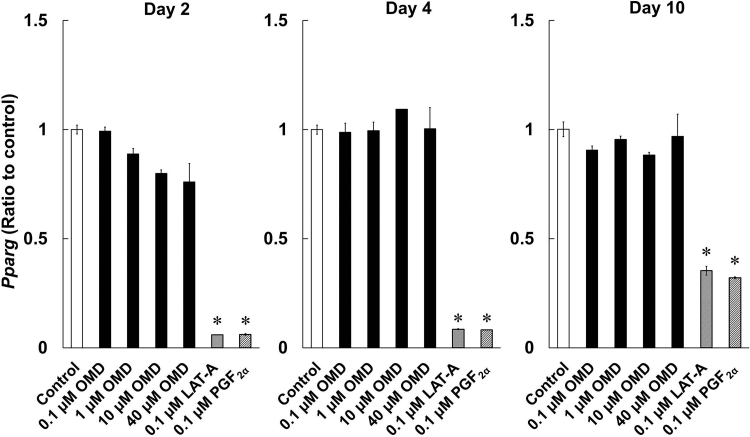
Effects of OMD and FP agonists on *Pparg* mRNA expression on days 2, 4, and 10 of differentiation. Differences in *Pparg* expression between time points were quantified by using real-time PCR. *P < 0.05, compared with control (Wilcoxon test).

**FIG. 7.**
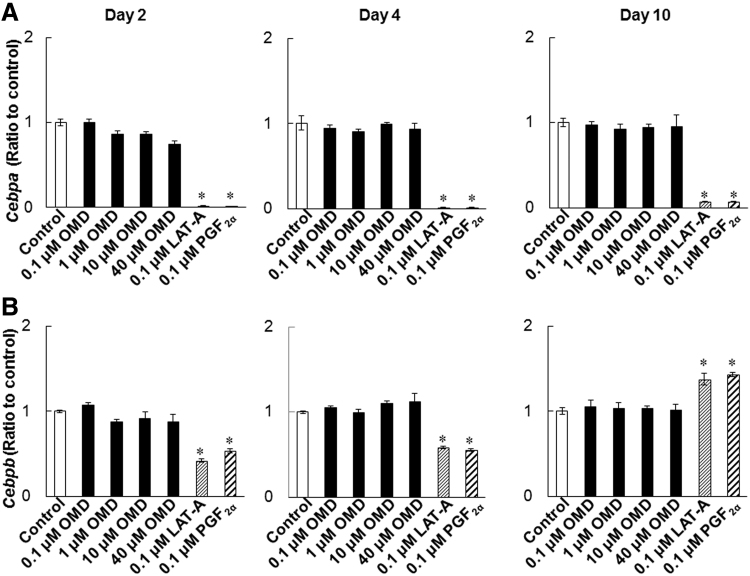
Effects of OMD and FP agonists on *Cebpa* and *Cebpb* expression levels during the differentiation. *Cebpa* **(A)** and *Cebpb* **(B)** expression levels on days 2, 4, and 10 were quantified by using real-time PCR. *P < 0.05, compared with control (Wilcoxon test).

To investigate the effects of OMD and FP agonists on adipogenesis, we determined *Pparg*, *Cebpa*, and *Cebpb* expression levels during differentiation of 3T3-L1 cells. LAT-A and PGF_2α_ suppressed the expression of *Pparg* and *Cebpa* on days 2, 4, and 10 (Figs. 6 and 7A). *Cebpb* expression levels were significantly lowered in LAT-A-and PGF_2α_-treated cells on days 2 and 4, compared with those in control differentiating cells (Fig. 7B). In contrast, even at 40 μmol/L, OMD did not affect expression levels of these genes under the present conditions.

## Discussion

In this study, we investigated the effects of the selective EP2 agonist OMD on adipogenesis and made comparisons with those of FP agonists in differentiating 3T3-L1 cells. OMD is a non-prostaglandin structure compound and OMDI, isopropyl ester of OMD, is an active pharmacological ingredient of ophthalmic solution for the treatment of glaucoma as an IOP-lowering agent.^17^ After topical administration to ocular surfaces, OMDI is hydrolyzed by esterases to OMD during its corneal penetration.^18^ During 2018, this drug was launched in Japan for the treatment of glaucoma and ocular hypertension.

It is reported that DUES is one of the cosmetic AEs induced by FP agonists and induced by atrophy of orbital fat in the eyelid by inhibiting adipogenesis.^21^ Although this AE was induced by stimulation of the FP receptor, no published studies demonstrate the effects of EP2 receptor agonists on adipogenesis. Overall, 3T3-L1 cells were originally developed by clonal expansion from murine Swiss 3T3 cells^27^ and have been widely used as preadipocytes in studies of adipocyte differentiation.^27–29^ In our hands, LAT-A and PGF_2α_ prevented the accumulation of lipid droplets in 3T3-L1 cells and inhibited adipogenesis (Figs. 3 and 4). In agreement, Taketani et al. showed that all FP agonists that are currently used as glaucoma treatments, including latanoprost, inhibited adipogenesis by stimulating the FP receptor in 3T3-L1 cells.^21^ In addition, FP agonists limited the induction of *Pparg*, *Cebpa*, and *Cebpb* during adipocyte differentiation (Figs. 6 and 7). Under normal conditions of adipogenesis, *Cebpb* is expressed early to transactivate *Pparg* and *Cebpa*, which are master transcriptional regulators of terminal adipocyte differentiation.^25,26^ Further, it is reported that differentiation starts with the induction of *Cebpb* at an early stage, during which cells begin to express *Pparg* and *Cebpa*.^28,29^ Our results are consistent with those of previous reports (Fig. 5).^28,29^ These observations suggest that FP agonists prevent the accumulation of lipid droplets initially by inhibiting adipogenic *Cebpb* expression, and subsequently by limiting the associated increases in *Pparg* and *Cebpa* expression.

A previous report suggested that C/EBPβ was involved in cell proliferation.^25^ We observed that *Cebpb* expression levels were significantly increased in LAT-A-treated cells on day 10, compared with those in control differentiating cells (Fig. 7B). The increase in cell viability in the LAT-A-treated group shown as in Fig. 2 may be due to the increase in *Cebpb* expression induced by LAT-A. *Cebpb* expression was also upregulated in the PGF_2α_-treated group (Fig. 7B), although the cell viability did not increase in that group (Fig. 2). This discrepancy may arise from the receptor selectivity of LAT-A and PGF_2α_.

In the present experiments, OMD did not affect lipid droplet accumulation or the expression levels of *Pparg*, *Cebpa*, and *Cebpb*, even at high concentrations of up to 40 μmol/L (Figs. 3,4,6, and 7). We also confirmed that EP2 and FP receptor mRNAs were expressed by 3T3-L1 cells under these conditions (data not shown), suggesting that OMD stimulates the EP2 receptor.

Activated FP receptor initiates several intracellular events, including signaling through the phospholipase C/IP_3_R/Ca^2+^ pathway.^30,31^ PGF_2α_ also inhibits adipocyte differentiation via the Gα_q_-Ca^2+^-calcineurin-dependent signaling pathway.^32^ Moreover, coupling of the EP2 receptor with G_s_ leads to elevated cAMP concentrations.^14^ Hence, multiple signaling pathways are involved in the downstream effects of FP and EP2 receptors, yet differences between these are considered central to the differing effects of the present receptor agonists on adipocyte differentiation. Previous report has shown that EP4 receptor stimulation increases cAMP and suppresses adipocyte differentiation,^33^ although EP4 signaling also plays Gα_i_-mediated roles that are independent of cAMP.^34^ Thus, the effects of EP4 receptor signaling on adipocyte differentiation may be mediated by additional cAMP independent mechanisms. Nonetheless, in contrast with EP4 agonists, the EP2 receptor agonist OMD did not suppress adipocyte differentiation, thus distinguishing the downstream effects of EP2 and EP4 receptors.

The mechanism of DUES induced by FP agonists remains unclear, although the prevention of adipogenesis is considered a potentially major cause, as supported by several studies.^12,21^ Choi et al. had reported that browning of adipocytes can be related to development of PAP.^35^ Using human orbital adipose tissue samples, they reported that bimatoprost upregulates pathways involved in the browning of adipocytes via MAPK, PI3/Akt, and p38.^35^ In addition, LAT-A activated the MAPKs extracellular signal-regulated kinase, p38, and c-Jun NH_2_-terminal kinase,^36^ indicating that FP agonist-induced PAP is also caused by browning of adipocytes via kinase pathways. In this study, we focused on the effect of OMD on adipogenesis and the regulation of its transcriptional factors (*Pparg*, *Cebpa*, and *Cebpb*) compared with those of FP agonists. However, we may need to investigate the effect of OMD on browning of adipocytes and its related kinase pathways to comprehensively examine the influence of OMD on PAP in future experiments.

In this study with 3T3-L1 cells, the highest dose of OMD (40 μmol/L) was equivalent to that of OMDI (0.002%) ophthalmic solution.^37^ OMD has strong agonistic activity and selectivity for the human EP2 receptor (hEP2 EC_50_ = 8.3 nmol/L).^18^ We confirmed these agonistic activities of OMD toward the mEP2 receptor (mEP2 EC_50_ = 3.9 nmol/L), and we showed that they are equivalent to those of hEP2 (Fig. 1C). Current results imply that OMDI ophthalmic solution has little to no effect on adipocyte differentiation in humans.

Our findings using the 3T3-L1 cell line may have limited the extrapolation to human periocular or orbital adipose tissues. A previous report by Choi et al. using biopsied human orbital adipose tissues indicated that *Pparg* and *Cebpa* contributed to their adipogenic differentiation.^38^ Further, they demonstrated that FP agonists inhibited the accumulation of intracytoplasmic lipid droplets by downregulation of *Pparg* and *Cebpa*, suggesting that FP agonists suppressed adipogenesis in human periocular and orbital adipose tissues *in vivo.*^38^ Similarly, in our study using the 3T3-L1 cell line, we observed upregulation of *Pparg* and *Cebpa* with adipogenic differentiation (Fig. 5), and they were significantly inhibited by FP agonists (Figs. 6 and 7), implying that there is at least a partial overlap between the factors involved in adipogenesis in human periocular and orbital adipose tissues and the 3T3-L1 cell line at the transcriptional level. Although further studies are needed to clarify the effects of OMD in human samples, we think that we can estimate the effects of OMD on adipocyte differentiation in humans in the 3T3-L1 cell line compared with FP agonists.

Long-term evidence in patients is needed, but current data suggest that OMDI does not induce DUES in glaucoma patients due to the different profile of OMD on gene expression related to adipogenesis in the eyelid fat tissue, unlike existing FP agonists.

## References

[1] Nucci C., Martucci A., Giannini C., Morrone L.A., Bagetta G., and Mancino R. Neuroprotective agents in the management of glaucoma. Eye 32:938–945, 2018 29472700 10.1038/s41433-018-0050-2 PMC5944652

[2] Weinreb R., Aung T., and Medeiros F. The pathophysiology and treatment of glaucoma. J. Am. Med. Assoc. 311:1901–1911, 2014 10.1001/jama.2014.3192 PMC4523637 24825645

[3] Klimko P.G., and Sharif N.A. Discovery, characterization and clinical utility of prostaglandin agonists for the treatment of glaucoma. Br. J. Pharmacol. 176:1051-1058, 2019 29665040 10.1111/bph.14327 PMC6451111

[4] Alm A., Grierson I., and Shields M.B. Side effects associated with prostaglandin analog therapy. Surv. Ophthalmol. 53(6 Suppl.):93–105, 2008 19038628 10.1016/j.survophthal.2008.08.004

[5] Patradul C., Tantisevi V., and Manassakorn A. Factors related to prostaglandin-associated periorbitopathy in glaucoma patients. Asia Pac. J. Ophthalmol. 6:238–242, 2017 10.22608/APO.2016108 28379653

[6] Kucukevcilioglu M., Bayer A., Uysal Y., and Altinsoy H.I. Prostaglandin associated periorbitopathy in patients using bimatoprost, latanoprost and travoprost. Clin. Exp. Ophthalmol. 42:126–131, 2014 23844550 10.1111/ceo.12163

[7] Ung T., and Currie Z.I. Periocular changes following long-term administration of latanoprost 0.005%. Ophthalmic Plast. Reconstr. Surg. 28:e42–e43, 2012 21659917 10.1097/IOP.0b013e31821d86a5

[8] Filippopoulos T., Paula J.S., Torun N., Hatton M.P., Pasquale L.R., and Grosskreutz C.L. Periorbital changes associated with topical bimatoprost. Ophthalmic Plast. Reconstr. Surg. 24:302–307, 2008 18645437 10.1097/IOP.0b013e31817d81df

[9] Inoue K., Shiokawa M., Wakakura M., and Tomita G. Deepening of the upper eyelid sulcus caused by 5 types of prostaglandin analogs. J. Glaucoma. 22:626–631, 2013 22936280 10.1097/IJG.0b013e31824d8d7c

[10] Nakakura S., Yamamoto M., Terao E., et al. Prostaglandin-associated periorbitopathy in latanoprost users. Clin. Ophthalmol. 9:51–56, 2014 25565768 10.2147/OPTH.S75651 PMC4284030

[11] Sakata R., Shirato S., Miyata K., and Aihara M. Incidence of deepening of the upper eyelid sulcus in prostaglandin-associated periorbitopathy with a latanoprost ophthalmic solution. Eye 28:1446–1451, 2014 25233818 10.1038/eye.2014.224 PMC4268464

[12] Park J., Cho H.K., and Moon J.I. Changes to upper eyelid orbital fat from use of topical bimatoprost, travoprost, and latanoprost. Jpn J. Ophthalmol. 55:22–27, 2011 21331688 10.1007/s10384-010-0904-z

[13] Sugimoto Y., and Narumiya S. Prostaglandin E receptors. J. Biol. Chem. 282:11613–11617, 2007 17329241 10.1074/jbc.R600038200

[14] Narumiya S., Sugimoto Y., and Ushikubi F. Prostanoid receptors: structures, properties, and functions. Physiol. Rev. 79:1193–1226, 1999 10508233 10.1152/physrev.1999.79.4.1193

[15] Stern F.A., and Bito L.Z. Comparison of the hypotensive and other ocular effects of prostaglandins E2 and F2 alpha on cat and rhesus monkey eyes. Invest. Ophthalmol. Vis. Sci. 22:588–598, 1982 7076404

[16] Matsou A., and Anastasopoulos E. Investigational drugs targeting prostaglandin receptors for the treatment of glaucoma. Expert Opin. Investig. Drugs. 27:777–785, 2018 10.1080/13543784.2018.1526279 30227753

[17] Iwamura R., Tanaka M., Okanari E., et al. Identification of a selective, non-prostanoid EP2 receptor agonist for the treatment of glaucoma: omidenepag and its prodrug omidenepag isopropyl. J. Med. Chem. 61:6869–6891, 2018 29995405 10.1021/acs.jmedchem.8b00808

[18] Kirihara T., Taniguchi T., Yamamura K., et al. Pharmacologic characterization of omidenepag isopropyl, a novel selective EP2 receptor agonist, as an ocular hypotensive agent. Invest. Ophthalmol. Vis. Sci. 59:145–153, 2018 29332128 10.1167/iovs.17-22745

[19] Fuwa M., Toris C.B., Fan S., et al. Effects of a novel selective EP2 receptor agonist, omidenepag isopropyl, on aqueous humor dynamics in laser-induced ocular hypertensive monkeys. J. Ocul. Pharmacol. Ther. 34:531–537, 2018 29989843 10.1089/jop.2017.0146

[20] Aihara M., Lu F., Kawata H., Iwata A., Odani-Kawabata N., and Shams N.K. Six-month efficacy and safety outcomes of a novel selective EP2 agonist omidenepag isopropyl: the RENGE study (phase 3). Invest. Ophthalmol. Visual Sci. 59:1229, 201829625443

[21] Taketani Y., Yamagishi R., Fujishiro T., Igarashi M., Sakata R., and Aihara M. Activation of the prostanoid FP receptor inhibits adipogenesis leading to deepening of the upper eyelid sulcus in prostaglandin-associated periorbitopathy. Invest. Ophthalmol. Vis. Sci. 55:1269–1276, 2014 24508785 10.1167/iovs.13-12589

[22] Miller C.W., Casimir D.A., and Ntambi J.M. The mechanism of inhibition of 3T3-L1 preadipocyte differentiation by prostaglandin F2alpha. Endocrinology. 137:5641–5650, 1996 8940395 10.1210/endo.137.12.8940395

[23] Katsuyama M., Nishigaki N., Sugimoto Y., et al. The mouse prostaglandin E receptor EP2 subtype: cloning, expression, and Northern blot analysis. FEBS Lett. 372:151–156, 1995 7556658 10.1016/0014-5793(95)00966-d

[24] Namba T., Sugimoto Y., Negishi M., et al. Alternative splicing of C-terminal tail of prostaglandin e receptor subtype EP3 determines G-protein specificity. Nature 365:166–170, 1993 8396726 10.1038/365166a0

[25] Guo L., Li X., and Tang Q.Q. Transcriptional regulation of adipocyte differentiation: a central role for CCAAT/enhancer-binding protein (C/EBP) β. J. Biol. Chem. 290:755–761, 2015 25451943 10.1074/jbc.R114.619957 PMC4294498

[26] Wedel A., and Lömsziegler-Heitbrock H.W. The C/EBP family of transcription factors. Immunobiology 193:171–185, 1995 8530141 10.1016/s0171-2985(11)80541-3

[27] Green H., and Kehinde O. Sublines of mouse 3T3 cells that accumulate lipid. Cell 1:113–116, 1974

[28] Dave S., Kaur N.J., Nanduri R., Dkhar H.K., Kumar A., and Gupta P. Inhibition of adipogenesis and induction of apoptosis and lipolysis by stem bromelain in 3T3-L1 adipocytes. PLoS One 7:e30831, 2012 22292054 10.1371/journal.pone.0030831 PMC3265525

[29] Diep D.T.V., Hong K., Khun T., et al. Anti-adipogenic effects of KD025 (SLx-2119), a ROCK2-specific inhibitor, in 3T3-L1 cells. Sci. Rep. 8:2477, 2018 29410516 10.1038/s41598-018-20821-3 PMC5802830

[30] Heaslip R.J., and Sickels B.D. Evidence that prostaglandins can contract the rat aorta via a novel protein kinase C-dependent mechanism. J. Pharmacol. Exp. Ther. 250:44–51, 1989 2545867

[31] Abramovitzs M., Boie Y., Nguyen T., et al. Cloning and expression of cDNA a for the human prostanoid FP receptor. J. Biol. Chem. 269:2632–2636, 1994 8300593

[32] Liu L., and Clipstone N.A. Prostaglandin F2α inhibits adipocyte differentiation via a Gαq-calcium-calcineurin-dependent signaling pathway. J. Cell. Biochem. 100:161–173, 2007 16888802 10.1002/jcb.21044

[33] Inazumi T., Shirata N., Morimoto K., Takano H., Segi-Nishida E., and Sugimoto Y. Prostaglandin E 2 -EP4 signaling suppresses adipocyte differentiation in mouse embryonic fibroblasts via an autocrine mechanism. J. Lipid Res. 52:1500–1508, 2011 21646392 10.1194/jlr.M013615 PMC3137015

[34] Yokoyama U., Iwatsubo K., Umemura M., Fujita T., and Ishikawa Y. The prostanoid EP4 receptor and its signaling pathway. Pharmacol. Rev. 65:1010–1052, 2013 23776144 10.1124/pr.112.007195

[35] Choi C.J., Tao W., Doddapaneni R., et al. The effect of prostaglandin analogue bimatoprost on thyroid-associated orbitopathy. Invest. Ophthalmol. Vis. Sci. 59:5912–5923, 2018 30551199 10.1167/iovs.18-25134 PMC6296211

[36] Liu Y., Liu Y., Xu D., and Li J. Latanoprost-induced cytokine and chemokine release from human Tenon's capsule fibroblasts: role of MAPK and NF-κB signaling pathways. J. Glaucoma. 24:635–641, 2015 25715004 10.1097/IJG.0000000000000140

[37] Duggan S. Omidenepag isopropyl ophthalmic solution 0.002%: first global approval. Drugs. 78:1925–1929, 2018 30465134 10.1007/s40265-018-1016-1

[38] Choi H.Y., Lee J.E., Lee J.W., Park H.J., and Jung J.H. In vitro study of antiadipogenic profile of latanoprost, travoprost, bimatoprost, and tafluprost in human orbital preadiopocytes. J. Ocul. Pharmacol. Ther. 28:146–152, 2012 22107041 10.1089/jop.2011.0160 PMC3315164

